# Research on ways to attract foreign patients to a Korean medicine clinic: a retrospective 2024 case study from Seoul

**DOI:** 10.3389/fmed.2025.1633139

**Published:** 2025-08-21

**Authors:** Jeong Hyeon Kim, Serin Lee, Mijoo Kim, Seung Hwan Lee, Jeong-Su Park

**Affiliations:** ^1^Tong-In Korean Medicine Clinic, Seoul, Republic of Korea; ^2^College of Korean Medicine, Dongguk University, Seoul, Republic of Korea; ^3^Department of Preventive Medicine, College of Korean Medicine, Semyung University, Jecheon-si, Republic of Korea

**Keywords:** medical tourism, Korean medical tourism, foreign patients, herbal medicines, traditional Korean medicines

## Abstract

**Introduction:**

This study aimed to analyze the clinical characteristics and treatment patterns of foreign patients visiting a single Korean medicine clinic to identify strategies for improving TKM-based medical tourism in South Korea.

**Methods:**

This study retrospectively reviewed the electronic medical records of 318 foreign outpatients who visited Tong-In Korean Medicine Clinic in Seoul from January to December 2024. Data on patient demographics, nationality, number of visits, treatment modalities, diagnoses, and herbal medicine prescriptions were collected and analyzed.

**Results:**

Three Hundred and Eighteen foreign patients visited a single Korean medicine clinic. 68.9% were female and 31.1% were male, with the majority in their 20s and 30s. Patients originated from 51 countries, with the highest proportions from the United States (31.45%), France (12.27%), and Singapore (8.49%). Most visits occurred in May and September. The most common type of case was musculoskeletal disorders (73, 9%). A total of 108 patients (34.0%) received combined modality treatment, with 88 patients (81.5%) receiving both acupuncture and internal medicine services. Herbal medicine was prescribed to 53.8% of patients, who took it for an average of 36.66 days per person. Pills were preferred over decoctions, with over twice as many patients taking pills (188) than decoctions (80).

**Discussion:**

The result of this study suggests that TKM-based medical tourism has a meaning in the realm of treating musculoskeletal disorders but also in internal medicine care. Also, this is the first post-COVID-19 study analyzing a TKM clinic in the context of medical tourism globalization. The growing demand for internal medicine services along with long-term herbal medicine use highlights the importance of integrated and personalized TKM medical programs to enhance the global competitiveness of TKM.

## 1 Introduction

Recently, there has been a growing trend of medical consumers seeking better quality healthcare services and economic benefits by traveling abroad, a phenomenon known as medical tourism (MT).

Medical tourism is an expanding multidisciplinary economic activity that integrates healthcare and tourism industries. According to a recent report, it is projected that medical tourism will experience significant growth, reaching approximately about 101.98 billion USDs by 2030 (1). Furthermore, medical tourism can function not only as a means to enhance individual health and wellbeing but also as a diplomatic and cultural tool to improve bilateral relations between countries (2).

Many countries such as Singapore, Thailand, and Malaysia (3) are actively investing in medical tourism as a key growth engine. Each country offers unique competitive advantages: Malaysia emphasizes affordability, Thailand is known for its extensive hospital infrastructure, and Singapore offers advanced medical technologies and highly skilled professionals. Reflecting this regional focus, recent studies had heavily focused on medical tourism in these countries. In 2024, Fauzi et al. (4) examined the knowledge structure of medical tourism particularly in Southeast Asia—identifying key thematic clusters, including service quality, economic contribution, and the roll of accreditation.

Korean medical tourism has also gained substantial attention internationally, bolstered by the global popularity of K-pop and the rise of K-beauty (2). This growing interest in Korean culture has created opportunities for cultural exchange and expands the appeal of Korea as a medical tourism destination. From ~248,000 foreign patients in 2022, the number surged to 606,000 in 2023, marking a significant increase. This figure surpassed the previous record set on 2019 (497,000 patients) by about 1.2 times. Notably, in 2024, the number of foreign patients dramatically raised to 1,170,478, achieving the highest figure since the launch of the foreign patient attraction initiative in 2009 (5). Given this surge in medical tourism, Traditional Korean Medicine (TKM) is emerging as a unique and distinctive feature of Korea's medical tourism strategy.

While the global use of Complementary and Alternative Medicine (CAM) is on the rise, CAM treatments are especially prevalent and institutionally integrated in East Asian countries. Nations such as South Korea, China, and Taiwan have adopted a dual or pluralistic medical system—doctors of both medicines practicing similar responsibilities and duties. It is important to understand that in Korea, TKM and western medicine (WM) coexist—each with independent, exclusive practice rights. Since the implementation of this dual medical system in 1951, South Korea has been among the first countries to cover TKM in its National Health Insurance (6). But unlike Chinese medical policy which pursued the integration of Traditional Chinese Medicine (TCM) and WM, in South Korea, WM and TKM exists parallel, physicians in each sector allowed to practice only within their licensed medical categories. Korean medicine doctors provide a wide range of therapeutic interventions—including acupuncture, moxibustion, herbal medicine, Chuna manual therapy, acupotomy, based on a holistic perspective. Korean medicine clinics and Korean medicine hospitals provide such medical services, and musculoskeletal disorders comprise a substantial proportion of outpatient cases. According to the 2024 national survey on the use of TKM, ~90% of outpatients visited TKM clinics for disease treatments, with 68.9% receiving care specifically for musculoskeletal conditions (7).

Foreign patients have increased across all medical specialties, with a particularly notable rise in the number of patients visiting Korean medicine clinics. In 2024, the number of patients visiting Korean medicine hospitals reached 33,893, a 84.6% increase compared to the previous year's figures (5). This ranked second among all specialties in term of year-on-year growth, highlighting the growing potential for TKM-based medical. Despite these increases, TKM still accounts for only about 2.7% of the total number of foreign patients, indicating significant room for further development. In contrast, TCM maintains a strong global dominance. According to China Daily (2017), about 200,000 foreign patients came to China annually for TCM treatment. Furthermore, as of 2023, the global TCM market size was valued to be approximately USD 60 billion, with projections reaching USD 98.3 billion by 2032 (8). Recent studies have also emphasized the strategic use of TCM in China's post-COVID-tourism recovery, demonstrating its role as a key attractor for inbound medical tourists (9). Given these developments, TKM holds considerable potential to grow alongside the rapidly expanding global market.

Since the 2009 revision of medical laws, South Korea has implemented various policies to promote TKM-based medical tourism. In March 2010, the Korean Medicine Medical Tourism Council was established, followed by the release of strategies to enhance Korean medicine medical tourism (10). Additionally, efforts have been made to integrate TKM treatments with wellness tourism and develop medical resource convergence programs as part of initiatives to globalize TKM (7). The Ministry of Health and Welfare also emphasized attracting foreign patients in the field of TKM as part of its 2023 strategy to invigorate foreign patient attraction, increasing the likelihood of Korean medicine becoming a high-value industry.

Particularly, TKM-based medical tourism in Korea is shifting focus from beauty or experience-oriented services to treatment-based healthcare services. Among patients visiting Korean medicine hospitals and clinics, the proportion of female patients is high (83.9%), with most patients in their 30s (29.5%), and a high percentage of outpatients (99.3%) (7). Treatments for spinal and musculoskeletal disorders have become popular among foreign patients, with acupuncture and herbal medicine treatments being the most preferred treatment methods (11). Patients from various countries, including Japan, Russia, and the United States, are increasingly seeking TKM treatments, opening the door for attracting patients from even more countries in the future.

Although several earlier studies have explored about TKM in medical tourism, there remains a significant lack of post-COVID-19 academic research. A 2013 study by Jaseng Korean Medicine Hospital on ways to attract foreign patients (11) highlighted that musculoskeletal disorders were the most common conditions treated among foreign patients, showing the potential for medical tourism focused on disease treatment rather than aesthetic purposes. Furthermore, a 2018 satisfaction research at the same hospital (12) revealed that 90.2% of foreign patients were satisfied, with 76.9% showing a positive shift in their perceptions of TKM treatments, suggesting a strong advantage in attracting foreign patients through TKM in medical tourism. Jaseng Korean Medicine Hospital, specializing in musculoskeletal disorders, had the highest number of foreign patients visiting via agencies in the 2018 study. Considering that Korean medicine clinics account for about 96% of the total Korean medicine institutions (14,736 Korean medicine clinics vs. 581 Korean medicine hospitals in 2024) (20), it is essential to include a wider range of Korean medicine institutions in future quantitative research. Also, due to the pandemic-induced disruption of research activities between 2020 and 2021, most recent publications have focused on governmental statistics and recovery trends rather than in-depth academic inquiry. As medical tourism began to recover in 2022, researches exploring TKM-based medical tourism have not been conducted. Therefore, in the post-COVID-19 context, it is essential to address this research gap.

There is also a lack of research on patient characteristics and treatment patterns, resulting in insufficient systematic discussion on effective ways to develop South Korea's TKM-based medical tourism. In light of this, future research should focus on in-depth studies and data-based improvement strategies to promote TKM-based medical tourism effectively.

Therefore, this study aims to analyze the patient characteristics and treatment patterns of foreign patients who visited Tong-In Korean Medicine Clinic in 2024, with a focus on identifying key factors that can effectively enhance the development of TKM-based medical tourism in South Korea. By examining demographic data, treatment modalities, and the use of herbal medicine, this research seeks to uncover valuable insights that can guide future strategies for promoting TKM in the global medical tourism market.

## 2 Methods

### 2.1 Study participants

This study included all 318 foreign outpatients who visited Tong-In Korean Medicine Clinic in Seoul for outpatient services from January 1, 2024, to December 31, 2024.

#### 2.1.1 Inclusion criteria

(1) All foreign patients recorded in the clinic's electronic medical records (EMR) as having visited in 2024, including individuals who were originally Korean nationals but had acquired foreign citizenship.

#### 2.1.2 Exclusion criteria

(1) Patients who were enrolled in the Korean National Health Insurance program.

### 2.2 Methods

This study employed a retrospective cross-sectional design, analyzing medical records collected over a 1-year period in 2024. The data were collected from the electronic medical records (EMRs) of the 318 foreign patients, including information on gender, age, nationality, date of first visit, treatment duration, number of visits, primary diagnoses, presenting symptoms, and types of treatments received. All data fields required for analysis were complete, and no missing data were identified.

While the study was limited to a single Korean medicine clinic, Tong-In Clinic was selected due to its active engagement with foreign patients, recording the highest number of foreign patient visits among Korean medicine clinics, excluding Korean medicine hospitals in South Korea. This allowed for a detailed post-COVID-19 foreign patient trend. However, we acknowledge the potential selection bias, and the findings may not be generalizable to all Korean medicine clinics nationwide.

### 2.3 Treatment modality and diagnoses

Diagnoses were standardized using KCD-8, which is based on ICD-10 and adapted for domestic use in Korea. Treatment modalities were categorized according to predefined classifications used in the clinic's EMR system, including acupuncture, moxibustion, cupping, herbal medicine, Chuna manual therapy, and pharmacopuncture.

### 2.4 Ethics, consent, and permissions

Regarding the collection and use of personal data, the study was approved by the Institutional Review Board (IRB) of Semyung University (SMU IRB 2025-01-001) as a retrospective analysis. All patient data were fully anonymized prior to analysis, ensuring that no individuals could be identified. Written informed consent was waived in accordance with applicable bioethics regulations and medical law.

## 3 Results

### 3.1 Gender and age analysis

**Figure 1:**
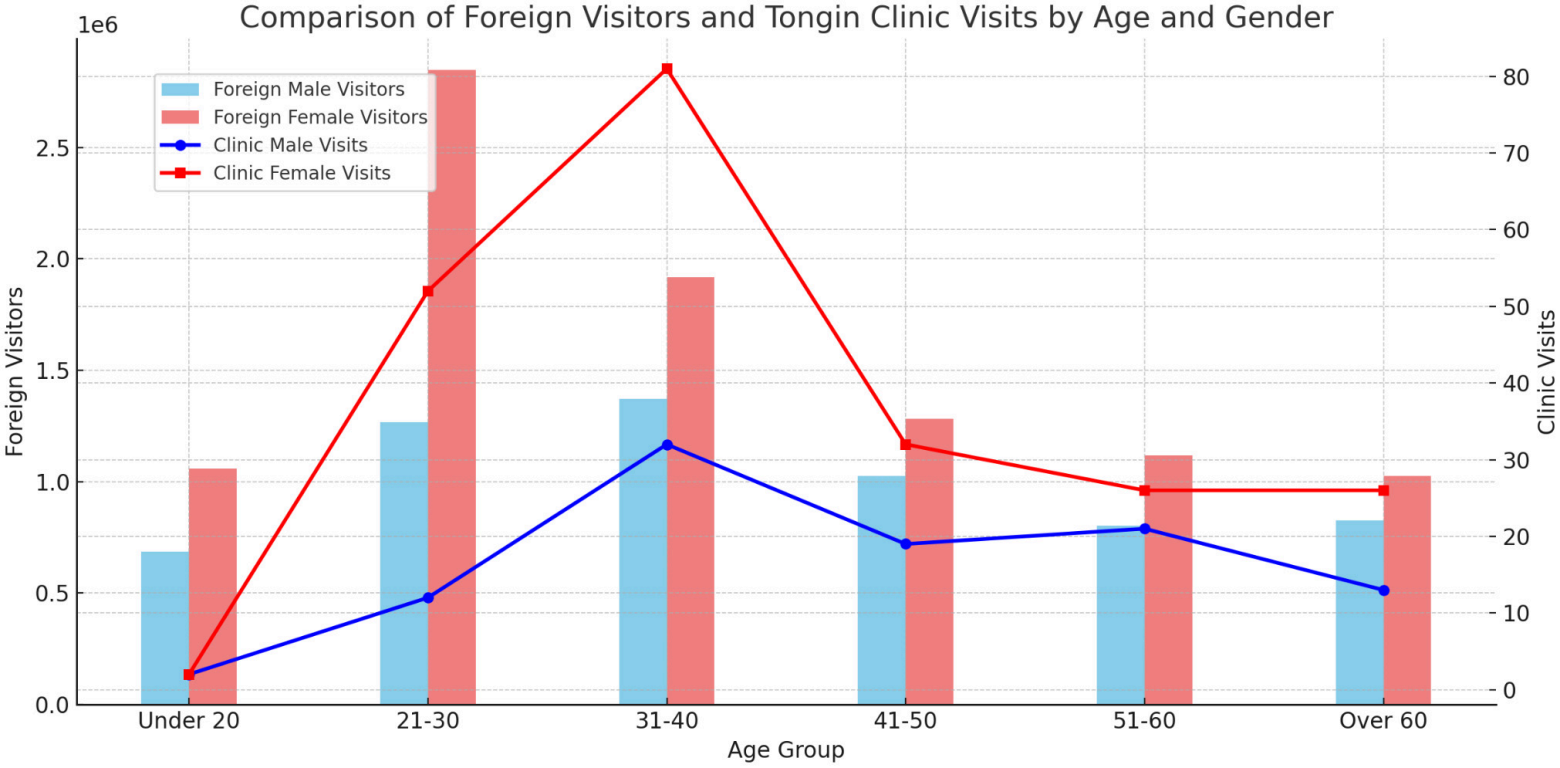
Comparison of foreign visitors and Tong-In Korean medicine clinic foreign patients by age and gender. Bar graph: Foreign Male Visitors and Foreign Female Visitors who visited South Korea in 2024. Line graph: TongIn Clinic's Foreign Male Visits and Foreign Female Visits in 2024 X-axis: age group; Y-axis: foreign visitors. Source: (13).

Among the 318 foreign patients, 219 (68.9%) were female and 99 (31.1%) were male, with females making up a higher proportion in all age groups. The largest group for both males and females was in their 30s, with ~32.32% of males and 36.99% of females in this age range. No patients in their 90s or older were observed (Figure 1).

### 3.2 Nationality analysis

**Figure 2:**
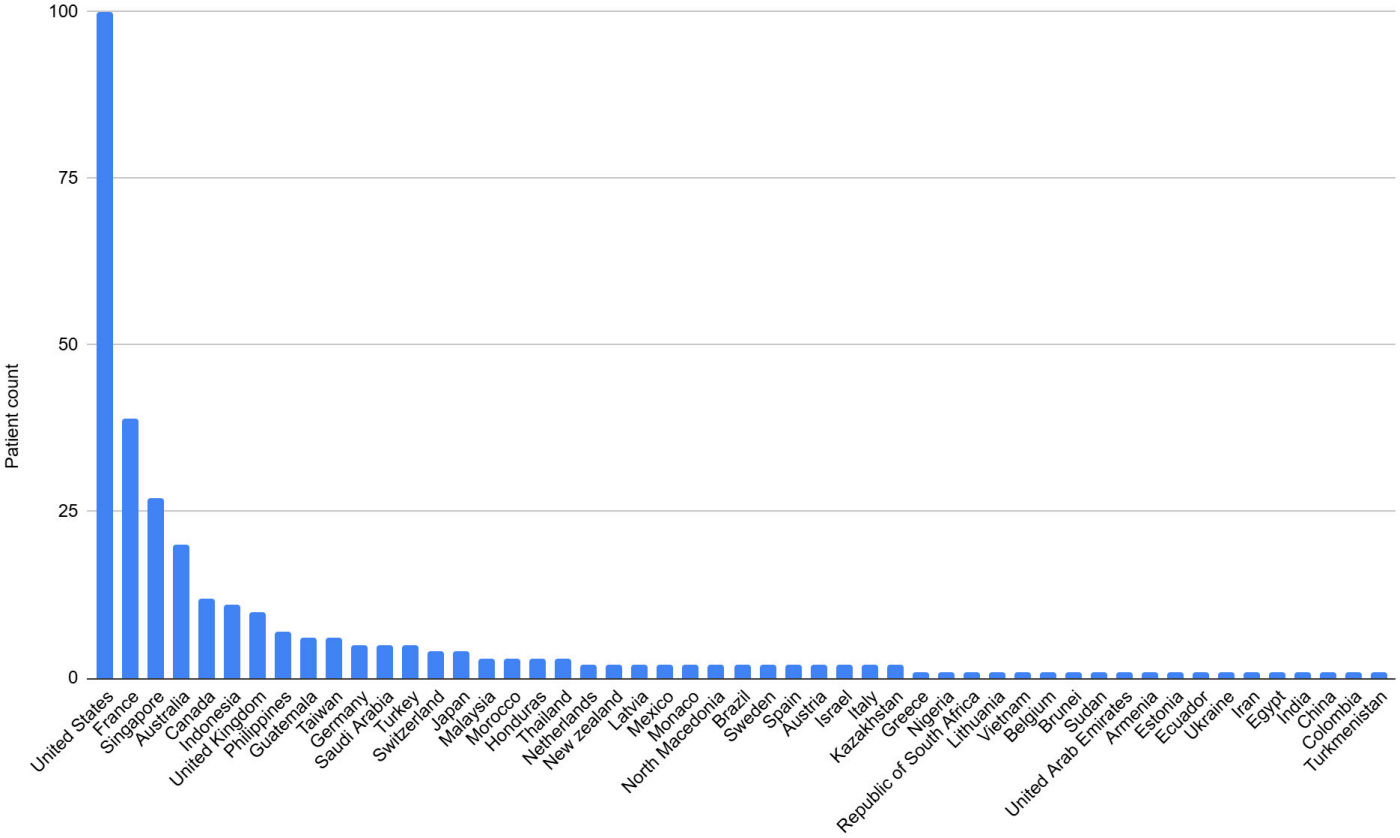
Distribution of foreign patients according to nationality.

**Figure 3:**
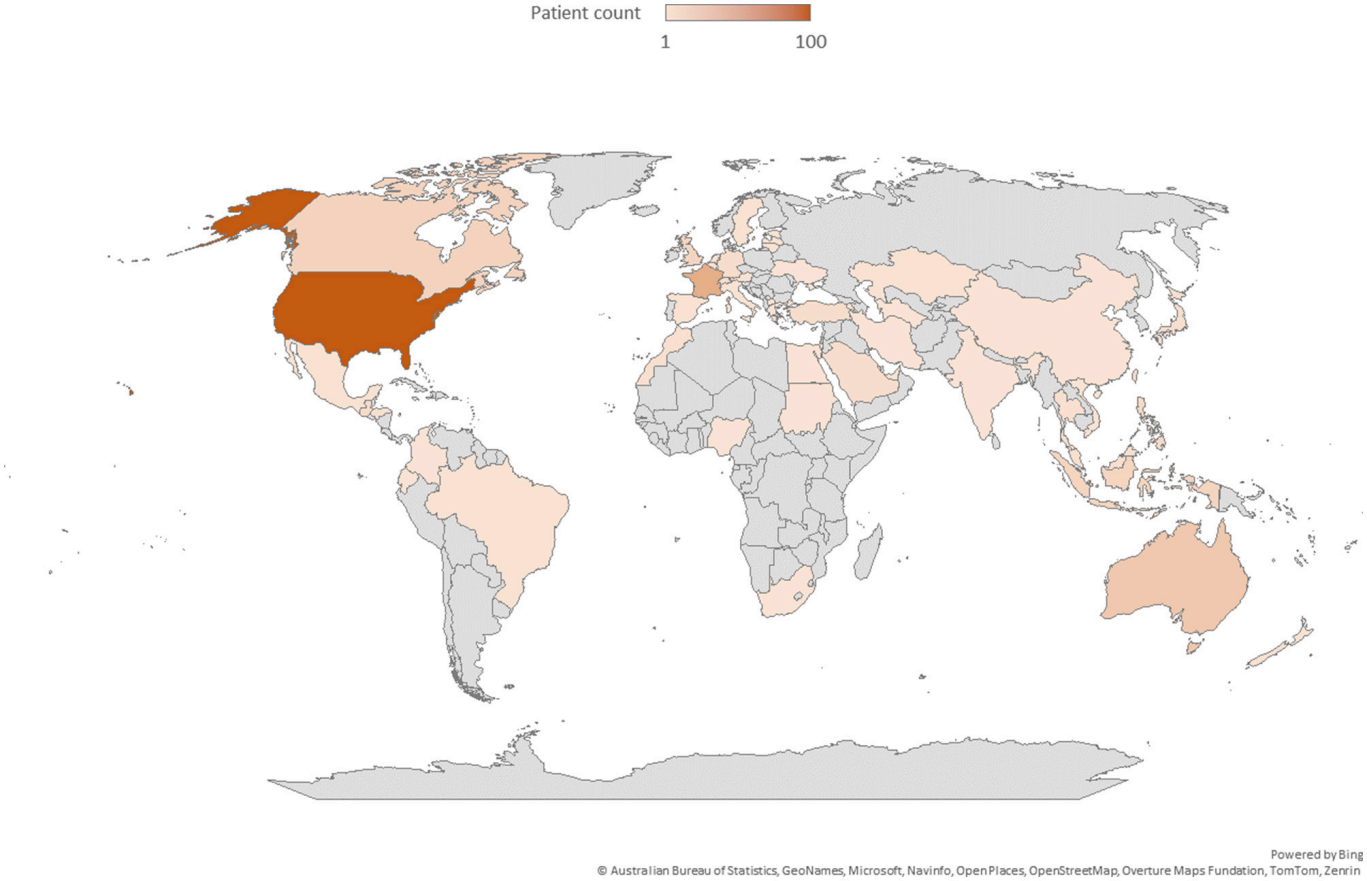
Distribution of foreign patients according to continent.

A total of 51 countries were represented among the foreign patients, with the highest proportions coming from the United States (31.45%), France (12.27%), and Singapore (8.49%). Notably, 53 patients visited from Southeast Asia, with Singapore accounting for half of these visits, indicating a significant concentration of patients from Singapore. This trend shows a higher percentage of English-speaking countries compared to East Asian countries (Figures 2, 3). In Table 1, countries with 3 or more patients were listed individually, while those with fewer than 3 patients were grouped and presented by continent (Table 1).

**Table 1:** Number of patients by country.

**Country**	***n***	**(%)**	**Continent**	***n***	**(%)**
United States	100	31.4	Southern Europe	7	2.2
France	39	12.3	Western Europe	7	2.2
Singapore	27	8.5	Central and South America	6	1.9
Australia	20	6.3	Northern Europe	6	1.9
Canada	12	3.8	Middle East	4	1.3
Indonesia	11	3.5	Central Asia	3	0.9
United Kingdom	10	3.1	Southeast Asia	2	0.6
Philippines	7	2.2	Oceania	2	0.6
Guatemala	6	1.9	North Africa	2	0.6
Taiwan	6	1.9	East Asia	1	0.3
Germany	5	1.6	Southwest Asia	1	0.3
Saudi Arabia	5	1.6	Eurasia	1	0.3
Turkey	5	1.6	Eastern Europe	1	0.3
Switzerland	4	1.3	West Africa	1	0.3
Japan	4	1.3	Southern Africa	1	0.3
Malaysia	3	0.9			
Morocco	3	0.9			
Honduras	3	0.9			
Thailand	3	0.9			

### 3.3 Monthly visit trends

**Figure 4:**
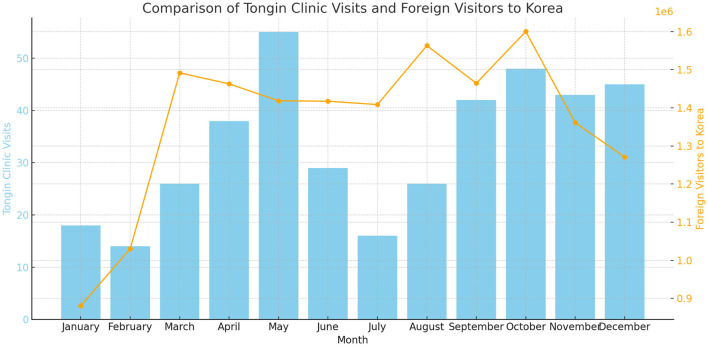
Comparison of Tong-In clinic visits and foreign visitors to Korea.

The highest number of visits occurred in May, with 55 patients. Visits were less frequent during the summer and winter, with consistent visits in spring and fall, especially in September through December (Figure 4).

To analyze whether the timing of foreign visitors in 2024 aligns with previous trends, we compared the global inbound tourism statistics from the Korean Tourism Organization's Tourism Knowledge Information System with the data from our clinic. The analysis revealed similar trends in the graph, indicating that clinic visits are also influenced by the characteristics of foreign tourists visiting South Korea.

### 3.4 Visit frequency and treatment duration

**Figure 5:**
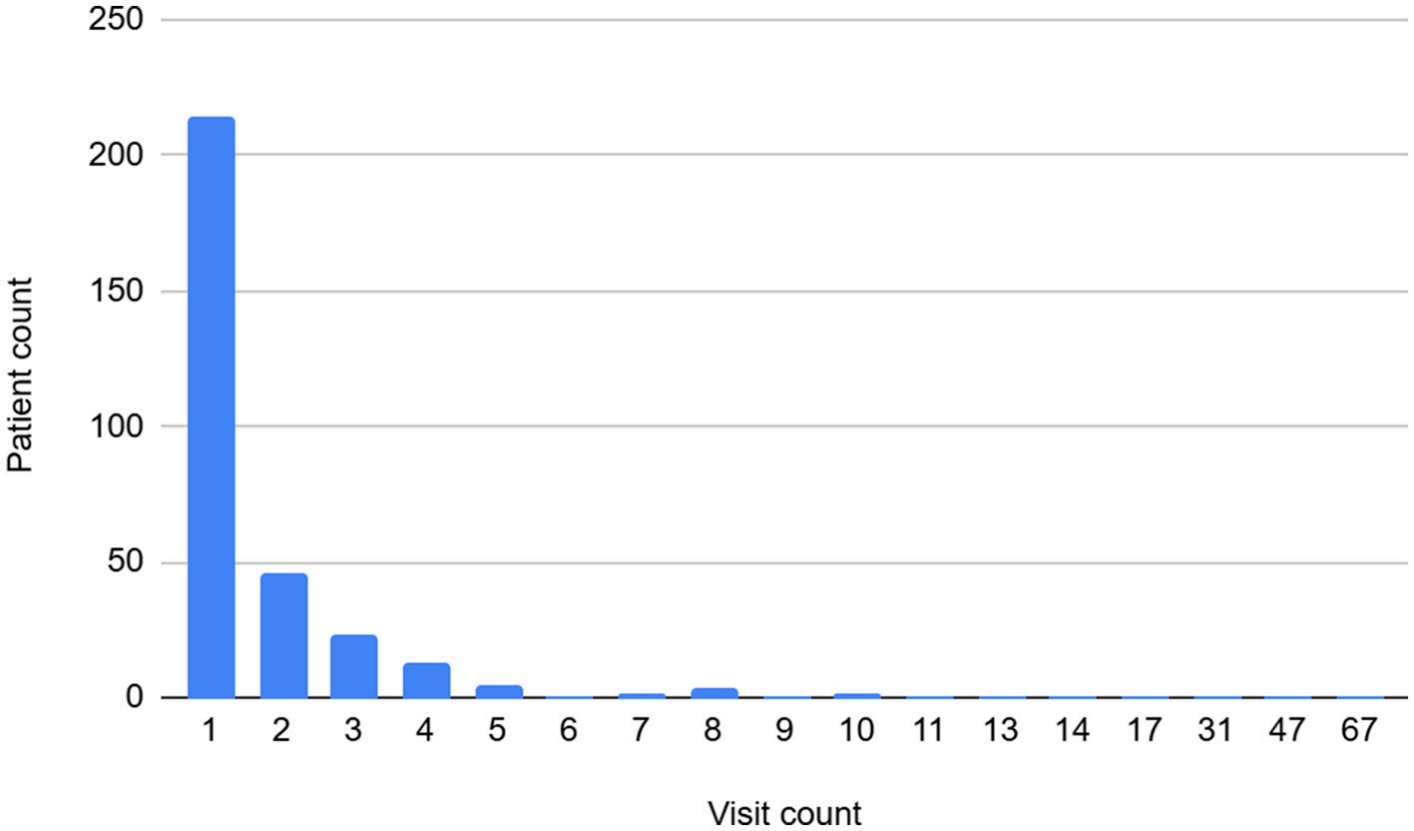
Distribution of foreign patient visits by frequency.

Patients varied in the number of visits, with 214 patients (67.3%) visiting only once. A small number of patients visited two or three times, reflecting the short-term visit nature of many medical tourists (Figure 5).

### 3.5 Treatment modality analysis

The treatment modality analysis was explained in Table 2.

**Table 2:** Analysis by department.

**Single department**	**Number of Patients (%)**
Acupuncture and Moxibustion Medicine	147 (71.0)
Internal Medicine of Korean Medicine	32 (15.5)
Neuropsychiatry of Korean Medicine	3 (1.4)
Obstetrics and Gynecology of Korean Medicine	8 (3.9)
Otorhinolaryngology of Korean Medicine	1 (0.5)
Ophthalmology of Korean Medicine	0 (0.0)
Rehabilitation Medicine of Korean Medicine	1 (0.5)
Dermatology of Korean Medicine	15 (7.2)
Other departments	3 (1.4)
Total	207 (100.0)
**Multiple departments**	**Number of patients**
Acupuncture and moxibustion medicine with another department	88 (81.5)
Two different departments	20 (18.5)
Total	108 (100.0)

#### 3.5.1 Treatment modality analysis

Out of the 318 patients, 207 patients (65.1%) received treatment in a single department. Among them, the highest number was treated in the acupuncture and moxibustion department, with 147 patients (70.9%), followed by the Korean medicine internal medicine department (32 patients, 15.5%), and Korean medicine dermatology (15 patients, 7.2%).

#### 3.5.2 Combined modality treatment

**Figure 6:**
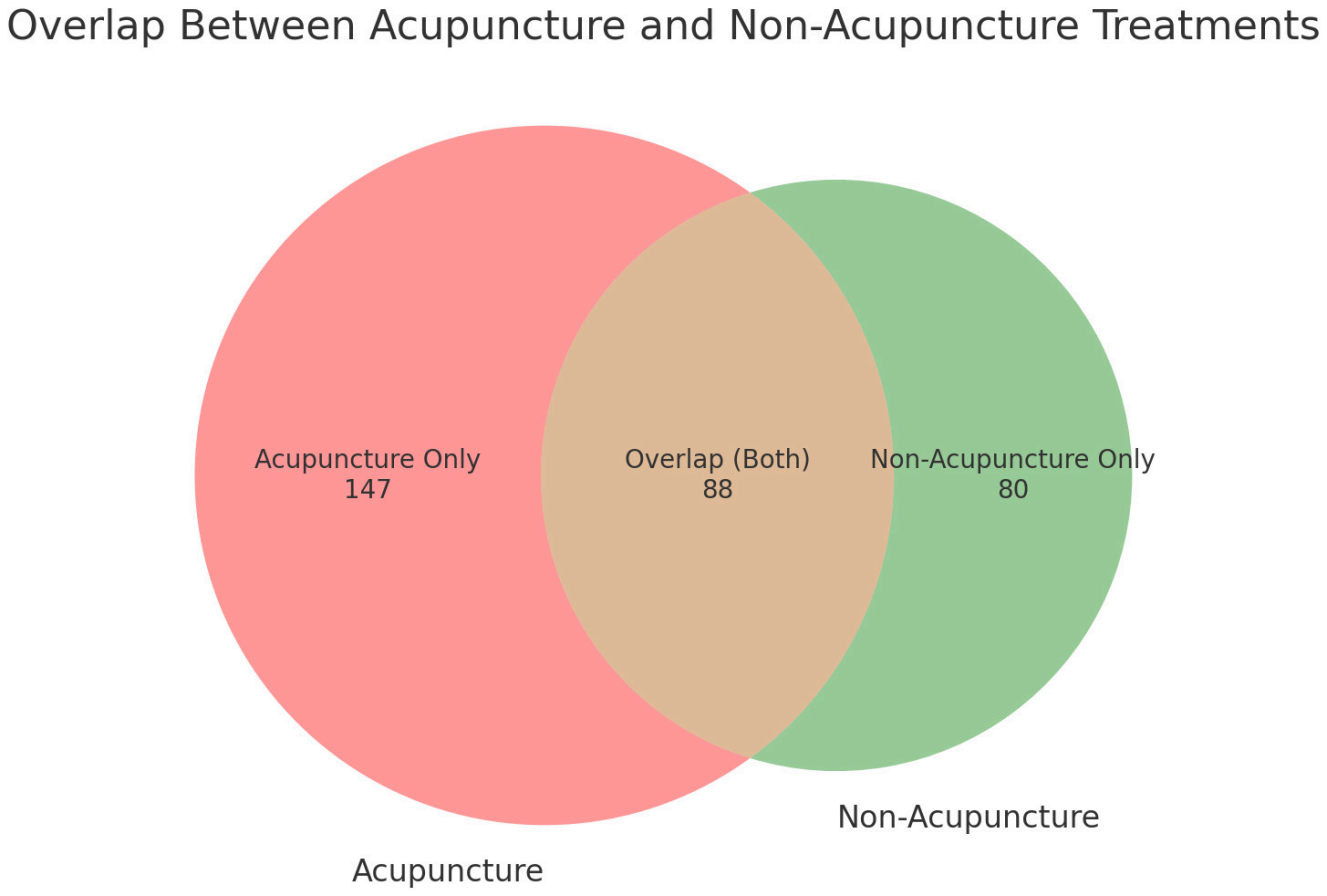
Overlap between acupuncture and non-acupuncture treatments.

A total of 108 patients (34.0%) received combined modality treatment. Among them, 88 patients (81.5%) received treatment in both the acupuncture and moxibustion department and another department (acupuncture + α) (Figure 6).

#### 3.5.3 Acupuncture and moxibustion

Of the 318 patients, 235 patients (73.9%) visited the acupuncture and moxibustion department for musculoskeletal disorders. A total of 147 patients (46.2%) received acupuncture and moxibustion treatment alone, while 88 patients (27.7%) received both acupuncture and moxibustion and internal medicine treatment.

#### 3.5.4 Non-acupuncture departments

Among all the patients, 88 patients (27.7%) received internal medicine treatment along with acupuncture and moxibustion treatment. Additionally, 80 patients (25.2%) received treatment solely for internal medicine conditions. In total, 168 patients (52.8%) received internal medicine treatment—indicating a growing trend of foreign patients seeking not only acupuncture but also internal medicine services.

### 3.6 Herbal medicine

One hundred seventy-one patients (53.8%) received herbal medicine prescriptions, with a preference for pills over decoctions. Due to overlapping prescriptions, 80 patients took decoctions while 188 took herbal pills, indicating a clear preference for pills—~2.3 times more patients chose pills over decoctions. During the same period, there were a total of 785 clinic visits and herbal medicine was prescribed in 326 cases (92 decoction prescriptions and 230 pill prescriptions), showing that pills were prescribed about 2.5 times more frequently than decoctions. Both the higher proportion of pill users among patients and the greater number of pill prescriptions suggest a strong patient preference for pills over decoctions. The average duration of herbal medicine use was 36.66 days per patient, with a mean prescription duration of 19.23 days per prescription suggesting a tendency toward long-term use.

#### 3.6.1 Frequency of prescriptions

Among the pill prescriptions, the most commonly prescribed formulas were Bangpungtongsung-hwan (22 patients), Gammaekdaejo-hwan (21 patients), and Yukmijihwang-hwan (16 patients) (Table 3). These formulas are primarily utilized for managing chronic conditions and improving constitutional health, suggesting their role in long-term therapeutic regimens. The top three decoction were Banhasashim-tang, Daehamhyoong-tang, each prescribed to 9 patients, followed by Ssangkum-tang, which was prescribed to 7 patients. These formulas are commonly used for digestive disorders, respiratory issues, and cold-related symptoms.

**Table 3:** Frequency of prescriptions.

**Frequency of prescriptions**			
**Decoction**	***N*** **(%)**	**Pills**	***N*** **(%)**
Banhasasim-tang 半夏瀉心湯	9 (11.3)	Bangpungtongsung-hwan 防風通聖丸	22 (11.7)
Daehamhyoong-tang 大陷胸湯	9 (11.3)	Gammaekdaejo-hwan 甘麥大棗丸	21 (11.2)
Ssangkum-tang 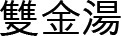	7 (8.8)	Yukmijihwang-hwan 六味地黃丸	16 (8.5)
Mahwang-tang 麻黃湯	6 (7.5)	Jakyakgamcho-hwan 芍藥甘草丸	15 (8.0)
Younggyechulgam-tang 苓桂朮甘湯	6 (7.5)	Palmijihwang-hwan 八味地黃丸	13 (6.9)
Pyungjin-tang 平陳湯	6 (7.5)	Ojeok-hwan 五積丸	12 (6.4)
Wolbigachul-tang 越婢加朮湯	4 (5.0)	Gyejibokryeong-hwan 桂枝茯苓丸	9 (4.8)
Jeongcheon-tang 定喘湯	3 (3.8)	Gwakhyanjeonggi-hwan 藿香正氣丸	8 (4.3)
Jakyakgamcho-tang 芍藥甘草湯	3 (3.8)	Bohwa-hwan 保和丸	7 (3.7)
Uhwangcheongsim-won (syr.) 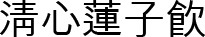 (湯濟)	3 (3.8)	Socheongryong-hwan 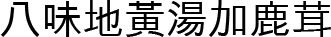	6 (3.2)
Maekmundong-tang 麥門冬湯	2 (2.5)	Seongjubogan-hwan 醒酒補肝丸	5 (2.7)
Jichul-tang 枳朮湯	2 (2.5)	Banhasasim-hwan 半夏瀉心丸	4 (2.1)
Sosiho-tang 小柴胡湯	2 (1.3)	Uhwangcheongsim-won 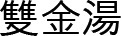	4 (2.1)
Yukmijihwang-tang 六味地黃湯	1 (1.3)	Dangguijakyak-hwan 當歸芍藥丸	4 (2.1)
Ojeok-san (syr.) 五積散 (湯濟)	1 (1.3)	Seonbangpaedok-hwan 仙防敗毒丸	4 (2.1)
Hyunburikyung-tang 玄附理經湯	1 (1.3)	Yoonjang-hwan 潤腸丸	4 (2.1)
Galgunhaegui-tang 葛根解肌湯	1 (1.3)	Cheonginyukwe-hwan 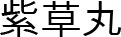	4 (2.1)
Shihogayonggolmoryo-tang 柴胡加龍骨牡蠣湯	1 (1.3)	Gongjin-dan 拱辰丹	4 (2.1)
Chijasi-tang 梔子鼓湯	1 (1.3)	Gamiheecheom-hwan 加味稀僉丸	3 (1.6)
Gyegal-tang 桂渴湯	1 (1.3)	Slim-hwan	3 (1.6)
Hwangryun-tang 黃連湯	1 (1.3)	Oryeong-hwan 五苓丸	3 (1.6)
Gigukyanghyeol-tang 杞菊養血湯	1 (1.3)	Haeulanshim-hwan 解鬱安心丸	3 (1.6)
Palmijihwang-tang (w/deer antler) 八味地黃湯加鹿茸	1 (1.3)	Youngsin-hwan 靈神丸	2 (1.1)
**Decoction**	***N*** **(%)**	**Pills**	***N*** **(%)**
Bangpungtongsung-san (syr.) 防風通聖散 (湯濟)	1 (1.3)	Chiljehyangbu-hwan 七製香附丸	2 (1.1)
Gwakhyanjeonggi-san (syr.) 藿香正氣丸 (湯濟)	1 (1.3)	Pyeongwi-hwan 平胃丸	2 (1.1)
Saengjimaekdong-eum 生地麥冬飮	1 (1.3)	Gyeongokgo-hwan 瓊玉膏丸	1 (0.5)
Jokyeongjongok-tang 調經種玉湯	1 (1.3)	Bojungikgi-hwan 補中益氣丸	1 (0.5)
Gyukhachukeo-tang 膈下逐瘀湯	1 (1.3)	Gwamin-jeon 過敏煎	1 (0.5)
Daeseunggi-tang 大承氣湯	1 (1.3)	Jacho-hwan 紫草丸	1 (0.5)
Younggyechulgam-tang (w/deer antler) 苓桂朮甘湯加鹿茸	1 (1.3)	Gamsu-hwan 甘遂丸	1 (0.5)
Palmijihwang-tang (w/deer antler) 八味地黃湯加鹿茸	1 (1.3)	Donggwajagagam 冬瓜子加減方	1 (0.5)
		Cheongsimyeonja-eum 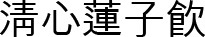	1 (0.5)
		Hangam-dan 抗癌丹	1 (0.5)
Total	80 (100.0)	Total	188 (100.0)

#### 3.6.2 Prescription by treatment department

Among the 215 patients treated, 24.19% were prescribed internal medicine, representing the largest proportion, followed by acupuncture and moxibustion (14.88%, 32 patients), Korean medicine neuropsychiatry (13.49%, 29 patients), and Korean medicine nephrology (13.02%, 28 patients). The Korean medicine obstetrics and gynecology department accounted for 11.16% (24 patients), while the Korean medicine respiratory department had 6.98% (15 patients), Korean medicine otorhinolaryngology was 6.05% (13 patients), Korean medicine rehabilitation medicine was 4.19% (9 patients), and Korean medicine dermatology was 3.26% (7 patients). The Korean medicine hepatology and cardiology departments each accounted for 2.79% (6 patients), representing the lowest proportions (Table 4).

**Table 4:** Prescription counts by department.

**Prescriptions by department**	**Number of patients**
Internal medicine of Korean medicine	107 (48.2)
1) Liver system	6
2) Cardiology and neurology	6
3) Digestive diseases	52
4) Lung system	15
5) Nephro-endocrine system	28
Acupuncture and moxibustion medicine	32 (14.4)
Neuropsychiatry of Korean medicine	29 (13.1)
Obstetrics and gynecology of Korean medicine	24 (10.8)
Rehabilitation medicine of Korean medicine	9 (4.1)
Ophthalmology, otorhinolaryngology, and dermatology of Korean medicine	21 (9.5)
1) Otorhinolaryngology	13
2) Ophthalmology	1
3) Dermatology	7
	222 (100.0)

#### 3.6.3 Prescription duration and frequency

Analysis of the total prescription duration and frequency revealed that Bangpungtongsung-hwan had the highest prescription frequency, with a total of 789 days and 28 prescriptions. It was followed by Palmijihwang-hwan with 687 days and 18 prescriptions, Yukmijihwang-hwan/Decoction with 436 days and 22 prescriptions, Gammaekdaejo-hwan with 376 days and 24 prescriptions, and Uhwangcheongsim-won/Decoction with 302 days and 13 prescriptions.

The average prescription duration per prescription was longest for Chijasi-tang, with an average of 90 days, followed by Yoonjang-hwan and Bojungikgi-hwan, each with 45 days. However, it is important to note that Chijasi-tang was prescribed to a single individual, thereby introducing potential bias that warrants careful consideration in the interpretation of these results. In contrast, Gyegal-tang had the shortest average prescription duration, with just 1 day of prescription (Table 5).

**Table 5:** Duration and frequency of drug administration.

**Prescription**	**Total prescription days (days)**	**Total prescription count (times)**	**Average prescription days per prescription**
Bangpungtongsung-hwan/tang 防風通聖丸/湯	789	28	28.2
Palmijihwang-hwan/tang 八味地黃丸/湯	687	18	38.2
Yukmijihwang-hwan/tang 六味地黃丸/湯	436	22	19.8
Gammaekdaejo-hwan 甘麥大棗丸	376	24	15.7
Uhwangcheongsim-won/syr. 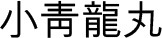	302	13	23.3
Chijasi-tang 梔子鼓湯	275	3	91.7
Ojeok-san/syr. 五積散/湯濟	256	13	19.8
Gyejibokryeong-hwan 桂枝茯苓丸	244	10	24.4
Jakyakgamcho-hwan 芍藥甘草丸	223	24	9.3
Slim-hwan	195	5	39
Gwakhyanjeonggi-hwan/tang 藿香正氣丸/湯	208	12	17.3
Yoonjang-hwan 潤腸丸	180	4	45
Gyeongokgo-hwan 瓊玉膏丸	179	5	35.8
Dangguijakyak-hwan 當歸芍藥丸	134	4	33.5
Banhasasim-tang 半夏瀉心湯	130	13	10
Bohwa-hwan 保和丸	128	9	14.2
Seongjubogan-hwan 醒酒補肝丸	128	5	25.6
Youngsin-hwan 靈神丸	120	3	40
Bojungikgi-hwan 補中益氣丸	90	2	45
Socheongryong-hwan 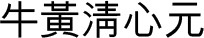	87	6	14.5
Haeulanshim-hwan 解鬱安心丸	74	4	18.5
Daehamhyoong-tang 大陷胸湯	71	10	7.1
Cheonginyukwe-hwan 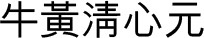	70	4	17.5
Pyeongwi-hwan 平胃丸	67	2	33.5
Palmijihwan-tang (w/deer antler) 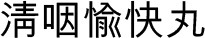	60	2	30
Donggwajagagam 冬瓜子加減方	60	1	60
Gongjin-dan 拱辰丹	49	5	9.8
Mahwang-tang 麻黃湯	38	7	5.4
Chiljehyangbu-hwan 七製香附丸	37	2	18.5
Oryeong-hwan 五苓丸	34	3	11.3
Pyungjin-tang 平陳湯	33	7	4.7
Younggyechulgam-tang 苓桂朮甘湯	31	8	3.9
Ssangkum-tang 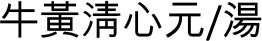	31	7	4.4
Jacho-hwan 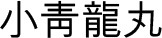	30	1	30
Hyunburikyung-tang 玄附理經湯	30	1	30
Younggyechulgam-tang (w/deer antler) 苓桂朮甘湯加鹿茸	30	1	30
Hwangryun-tang 黃連湯	30	1	30
Seonbangpaedok-hwan 仙防敗毒丸	27	5	5.4
Cheongsimyeonja-eum 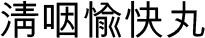	18	2	9
Galgunhaegui-tang 葛根解肌湯	18	2	9
Sosiho-tang 小柴胡湯	16	2	8
Shihogayonggolmoryo-tang 柴胡加龍骨牡蠣湯	14	1	14
Gamiheecheom-hwan 加味稀僉丸	9	3	3
Maekmundong-tang 麥門冬湯	9	2	4.5
Wolbigachul-tang 越婢加朮湯	8	4	2
Jeongcheon-tang 定喘湯	9	3	3
Jichul-tang 枳朮湯	6	2	3
Hangam-dan 抗癌丹	5	1	5
Gamsu-hwan 甘遂丸	4	1	4
Gwamin-jeon 過敏煎	3	1	3
Gigukyanghyeol-tang 杞菊養血湯	60	2	30
Saengjimaekdong-eum 生地麥冬飮	15	1	15
Jokyeongjongok-tang 調經種玉湯	15	1	15
Gyukhachukeo-tang 膈下逐瘀湯	30	1	30
Daeseunggi-tang 大承氣湯	30	1	30
Gyegal-tang 桂渴湯	1	1	1

## 4 Discussion

Before the COVID-19 pandemic, research on activating TKM-based medical tourism focused on various strategies to attract foreign patients. In particular, a study by Jaseng Korean Medicine Hospital in 2018 (12) proposed integrating Korean medicine into the medical tourism model, highlighting areas for improvement and the direction Korean medicine treatments should take in attracting foreign patients. However, following the pandemic in 2020, medical tourism experienced stagnation, necessitating a new approach.

Additionally, recent studies on TKM-based medical tourism were all conducted prior to COVID-19, meaning they do not reflect the changed dynamics of medical tourism in the post-COVID-19 era. Compared to pre-COVID-19 studies, this study highlights a shift in patient demographics and treatment preferences, suggesting a new direction for TKM-based medical tourism.

A study by Lee et al. (14) found that even foreign nationals residing in Korea had low awareness of TKM, with acupuncture primarily associated with pain and stress management. Factors inhibiting the use of TKM included lack of awareness, accessibility issues, doubts about certification, fear of needles, and a perceived lack of scientific evidence. Among 20 participants, 17 were familiar with CAM (complementary and alternative medicine), 16 with TCM (Traditional Chinese Medicine), and acupuncture, but only 7 were familiar with TKM (Traditional Korean Medicine). Furthermore, only 9 participants had experienced acupuncture, and 8 had tried herbal medicine. This indicates that even long-term foreign residents in Korea have low recognition of TKM. Therefore, improving awareness and accessibility of TKM, along with presenting scientific evidence, will be essential strategies for revitalizing medical tourism post-COVID-19.

In this context, it is crucial to analyze the characteristics and treatment patterns of foreign patients in depth to establish effective strategies for attracting them. This study comprehensively analyzed the gender, age, nationality, visit times and frequency, disease types, and herbal medicine usage of foreign patients.

The analysis of patient gender and age distribution revealed that the highest proportion was women in their 20s and 30s (Korea Tourism Organization). This trend mirrors the gender and age distribution of foreign tourists visiting Korea in 2024. A 2024 study by Lee and Ghangalso found that foreign women visited outpatient services more often than men, with the largest age group being 20–34 years (36.2%) (21). This contrasts with the higher use of TKM by older domestic patients (86.6% for those over 60) (15). These results suggest that TKM-based medical tourism should target foreign women in their 20s and 30s, rather than focusing solely on older age groups.

To establish a positive image of TKM treatments among foreign female tourists, it will be crucial to provide systematic information and strengthen the reliability of medical services. Foreign patients visited from 51 different countries, with the largest proportions coming from the United States (31.45%), France (12.27%), and Singapore (8.49%). Notably, 53 patients came from Southeast Asia, with Singapore accounting for half of them, indicating a trend of concentrated visits from certain countries. However, this differs from the nationality ranking of foreign tourists visiting Korea in 2024, where East Asian countries such as China, Japan, and Taiwan were the largest contributors. This suggests that patients from East Asian countries may already have access to TCM at home, reducing their need for TKM services. Therefore, it is essential to raise awareness of TKM services in East Asian countries like China, Japan, Taiwan, and Hong Kong. For countries with higher visitation rates like the United States, France, and Singapore, continued networking and tailored medical tourism packages will be crucial for maintaining a loyal patient base (16).

Seasonal trends in patient visits showed that foreign patients visited most frequently in May and from September to December, with a noticeable decrease during the winter months. This aligns with the seasonal patterns observed in foreign tourist visits in 2024 (Korea Tourism Organization). Therefore, offering specialized programs and promotions during peak months could be effective in attracting more patients.

Analysis of the visit frequency revealed that 214 patients (67.3%) visited only once, reflecting the short-term nature of medical tourism. To encourage repeat visits and create positive experiences, it will be important to ensure that patients experience high treatment effectiveness within a short period during their initial visit. Among the patients, 65.1% received single modality treatment, while 34% received combined modality treatment. The highest percentage of patients (70.9%) received treatment in the acupuncture and moxibustion department alone, with 73.9% of these patients seeking treatment for musculoskeletal disorders. This aligns with previous studies indicating that musculoskeletal disorders and acupuncture treatments are the most preferred types of care in TKM-based medical tourism. However, it is worth noting that many patients who visited the acupuncture department also received internal medicine treatment, with 81.5% of combined modality patients receiving acupuncture alongside non-acupuncture treatments. Among the 171 patients (53.8%) who received herbal medicine treatment, pills were preferred over decoctions due to convenience, with an average treatment duration of 36.66 days per patient and an average prescription duration of 19.23 days per prescription. This indicates a preference for long-term and systematic health management, rather than short-term symptom relief. Compared to a pre-COVID-19 research (12), several clear shifts in patient demographics, and treatment preferences were observed in this study. In terms of age distribution, the 2018 Jaseng study reported that the majority of foreign patients were in their 40s (26.7%), followed by those in their 50s (23.8%) and 30s (20.8%). In contrast, this study data shifts to a younger demographic—the largest age groups were those in their 30s (113 patients, 35.5%) and 20s (64 patients, 20.1%). Only 51 patients (16.0%) were in their 40s and 47 patients (14.8%) in their 50s. This suggests a reversal of the age trend observed in 2018, showing the growing interest in TKM among younger generations possibly influenced by increased exposure to Korean culture and wellness content online.

Also, the national origin of patients differed significantly between the two studies. The 2018 Jaseng study indicated that majority of patients came from Japan (26.9%), Russia (26.7%), and Kazakhstan (20.4%). However, in the present study, only four patients (0.3%) were from Japan, none from Russia, and two (0.6%) from Kazakhstan. Conversely, patients from the United States represented the highest proportion in this study, a stark contrast to their relatively small share (2.89%) in the Jaseng dataset. This discrepancy may be partly explained by the institutional characteristics of Jaseng Hospital, which is a spine-specialty Korean medicine hospital primarily focused on musculoskeletal disorders. In contrast, Tong-In Clinic is a single Korean medicine clinic which offers a broader range of treatments including internal medicine, gynecology, and general wellness care. This might attract a different patient demographic and explain the higher proportion of visitors from the US and other western countries.

Furthermore, there were differences in the types of treatment sought. In the 2018 study, most patients visited for musculoskeletal disorders and only 199 patients (11.48%) received herbal medicine prescriptions. The primary herbal formula prescribed was Chungpa-jun, specifically used for musculoskeletal conditions in Jaseng Hospital. In contrast, this study found that 171 out of 18 patients (53.8%) were prescribed herbal medicine, with a greater proportion of prescriptions issued for non-musculoskeletal conditions such as gynecological and digestive disorders. This suggests a broader utilization of herbal medicine in TKM clinics, reflecting a shift in patient needs and treatment orientation in the post-COVID-19 context.

These shifting trends suggest that foreign patients are interested not only in pain management treatments like acupuncture and Chuna therapy, but also in improving internal medicine symptoms such as digestive and circulatory issues. Therefore, for future development of TKM-based medical tourism, strategies that integrate acupuncture and moxibustion treatments with internal medicine treatments will be needed to provide comprehensive care for various conditions.

However, despite the high demand, there is still no official distribution channel for continuing herbal medicine use after foreign patients return to their home countries. A study by Labonté et al. (17) highlighted that follow-up care for patients after their return is a significant issue. Research by Jun et al. found that foreign patients who had received treatment at Korean medicine hospitals in Seoul preferred herbal medicine pills due to ease of storage and management. In medical tourism, where patient visits are often limited to one or two visits, establishing an efficient system for overseas delivery of herbal medicine and implementing follow-up care via remote systems will be crucial to ensuring continuity of treatment.

Establishing telemedicine partnerships with certified international pharmacies could further support the safe and legal overseas delivery of herbal medicines, addressing both continuity of care and regulatory compliance. For instance, China has seen rapid expansion of online pharmacies and internet hospital platforms that integrate telemedicine with pharmaceutical services. This aligns with recent global trends in digital pharmacy services, where online platforms are increasingly utilized not only for drug distribution but also for emerging areas such as cosmeceuticals and traditional medicine dispensing (18).

Currently, some Korean medicine clinics use private international shipping services, but there are concerns that herbal medicine may be mixed with non-professional health products, leading to potential quality, and safety issues. These concerns could damage the credibility of herbal medicine and negatively affect long-term patient management. Therefore, institutional improvements are essential to ensure the quality and safety of herbal medicine. It is also imperative from an economic standpoint. Yu and Choi (19) emphasized that remote healthcare and institutional improvements are necessary for securing a competitive position in the global medical tourism market. Countries with well-established medical tourism, such as Thailand, Singapore, and India, have implemented remote healthcare services to address declining patient margins. Comprehensive institutional improvements are expected to have positive effects on both patient demand and the economic aspect of the industry.

Thus, it is crucial for the TKM Association to collaborate with the Ministry of Health and Welfare and the Ministry of Foreign Affairs to develop and manage official overseas distribution channels and establish related institutional mechanisms. With these institutional improvements, TKM can enhance its credibility and accessibility for foreign patients, ultimately positioning itself as a key player in global wellness by expanding its scope from musculoskeletal treatments to internal medicine care.

Furthermore, the complexity of the registration process for medical institutions attracting foreign patients and the issues with maintaining these registrations has been problematic for healthcare providers. Simplifying administrative procedures and offering multifaceted support for registered medical institutions will foster a more proactive environment for Korean medicine clinics to attract foreign patients.

The absence of TKM from the main menu of the Medical Korea website, which operates to attract foreign patients, indicates that the Korean medicine sector is still sidelined in the medical tourism field. As of March 2025, only 967 Korean medical facilities (831 clinics, 136 hospitals) (22) are registered as medical institutions for foreign patient attraction, compared to the total of 15,317 Korean medical facilities (14,736 clinics, 581 hospitals) (20), which accounts for just 6.31% of the total. This suggests that TKM has not received sufficient promotion and policy support for foreign patient attraction. To address this, inclusion of TKM services in the main interface of South Korea's “Medical Korea” portal should be necessary to improve international visibility and accessibility.

While this study provides meaningful insights as the first clinic-based analysis of TKM for foreign patients, it is limited by its single-clinic data source, which may not reflect broader national trends. Furthermore, this study did not capture patient satisfaction or clinical outcome data such as treatment efficacy, which limits assessment of the actual impact of TKM treatments on foreign patients.

This study confirms that TKM-based medical tourism is expanding beyond the treatment of musculoskeletal disorders to include internal medicine conditions. Given that the highest proportion of patients were women in their 20s and 30s, marketing and policies for TKM-based medical tourism should target this demographic. Institutional improvements are necessary to integrate TKM more actively into medical tourism policies and support its development. Continuous research and policy support are essential for ensuring that TKM remains competitive in the global medical market.

## 5 Conclusion

This study analyzed 318 foreign patients who visited a single Korean medicine clinic in Seoul in 2024 and reached the following conclusions:

1. Among all foreign patients, 219 were female and 99 were male, with females making up a higher proportion in all age groups. Both male and female patients in their 30s were the largest group, with ~32.32% of males and 36.99% of females in this age group.
2. The most common nationalities were from the United States, France, and Singapore, which accounted for 52.2% of all foreign patients. A total of 53 patients came from Southeast Asia, and the number of patients from East Asian countries such as China, Japan, Taiwan, and Hong Kong were relatively low.
3. Foreign patients tended to visit between May and December, with a sharp decline in winter. The highest number of visits was for a single visit, with 214 patients (67.3%) visiting only once.
4. 73.9% (235 patients) of all patients visited for musculoskeletal disorders in the acupuncture and moxibustion department, with 46.2% (147 patients) receiving acupuncture and moxibustion treatment alone and 27.7% (88 patients) receiving both acupuncture and moxibustion and internal medicine treatments.
5. 53.8% (171 patients) of patients received herbal medicine treatment, with a preference for pills (96 cases) over decoctions (230 cases). The average duration of herbal medicine use was 36.66 days, and the average duration per prescription was 19.23 days. Many patients who received multi-departmental treatments also received herbal medicine treatment, with some receiving two or more prescriptions.

These findings reflect a shift from pre-COVID-19 trends, showing growing interest in TKM among younger, Western patients which suggests a broader role for TKM in holistic health and wellness tourism. To meet these evolving needs, policy efforts should support the development for short-term intensive care programs, simplification of registration procedures for foreign patient attraction, and improve systems for herbal medicine delivery abroad. Being among the first post-COVID-19 studies on TKM medical tourism, we hope that this research provides timely insights and may inform future strategies to attract foreign patients. Future research should explore trends across different TKM clinics and examine long-term treatment outcomes among international patients as this study is limited by its single-clinic data source.

## Data Availability

The data analyzed in this study is subject to the following licenses/restrictions: the dataset from medical record of clinic. Requests to access these datasets should be directed to Seung Hwan Lee, wooricare@naver.com.

## References

[1] Grand View Research. Medical tourism market size, share and trends analysis report by treatment type, by service provider, by country, and segment forecasts, 2025–2030 (2024). Available online at: https://www.grandviewresearch.com/industry-analysis/medical-tourism-market (Accessed June 23, 2025).

[2] Kim HL Hyun SS. The future of medical tourism for individuals' health and well-being: a case study of the relationship improvement between the UAE (United Arab Emirates) and South Korea. Int J Environ Res Public Health. (2022) 19:5735. 10.3390/ijerph19095735 35565130 PMC9104082

[3] Md Zain NA Abd Mutalib W Hanafiah MH Mohd Zahari MS Asyraff MA. Exploring medical tourism competitiveness in Malaysia, Thailand, and Singapore. World J Adv Res Rev. (2024) 24:44–52. 10.21837/pm.v21i30.1403

[4] Fauzi MA Aripin NM Alimin NSN Ting IWK Wider W Maidin SS. Medical tourism in South East Asia: science mapping of present and future trends. Asian Educ Dev Stud. (2024) 13:393–411. 10.1108/AEDS-04-2024-0093

[5] Ministry of Health and Welfare. Korea attracts 1.17 million foreign patients in 2024, breaking all-time record [Press release]. Ministry of Health and Welfare of South Korea (2025). Available online at: https://www.mohw.go.kr (Accessed June 24, 2025).

[6] Lee T. The integration of Korean medicine in South Korea. Acupunct Med. (2015) 33:96–7. 10.1136/acupmed-2015-010796 25762801

[7] Ministry of Health and Welfare. Announcement of 2023 foreign patient attraction performance [Press release]. Ministry of Health and Welfare of South Korea (2024). Available online at: https://www.mohw.go.kr/ (Accessed April 15, 2025).

[8] Traditional Chinese Medicine Market. Global Traditional Chinese Medicine Market Report (2024). Available online at: https://dataintelo.com/report/global-traditional-chinese-medicine-market (accessed June 21, 2025).

[9] Wen J Wang CC Goh E Su Z Ying T. Traditional Chinese medicine as a tourism recovery drawcard to boost China's inbound tourism after COVID-19. Asia Pac J Market Logist. (2022) 34:385–400. 10.1108/APJML-10-2020-0732

[10] Korea Health Industry Development Institute. Strategies for revitalizing international medical tourism. Korea Health Industry Development Institute Report (2010). Available online at: https://www.korea.kr/docViewer/skin/doc.html?fn=196140andrs=/docViewer/result/2010.03/16/19614

[11] Jun JY Nam JH Lee MJ Kim KW Lim SJ Lee CW. Research on ways to attract foreign patients based on analysis of foreign patients who visited hospital of Korean medicine. J Acupunct Res. (2013) 30:107–14. 10.13045/acupunct.2013027

[12] Shin J Lee YJ Shin JS Lee J Kim H Kim MR. Utilization status and satisfaction with medical services in nonresidential foreign medical tourists visiting a Korean medicine hospital. Evid Based Complement Alternat Med. (2018) 2018:6586352. 10.1155/2018/6586352 29853966 PMC5960560

[13] Korea Tourism Organization. Tourism Knowledge Information System. Korea Tourism Data Lab (n.d.). Avialable online at: https://datalab.visitkorea.or.kr/datalab/portal/nat/getForTourDashForm.do (Accessed April 15, 2025).

[14] Lee SM Park I Miller DB Lee S. Perceptions and experiences of acupuncture among expatriates living in Korea: a qualitative study. Korean J Acupunct. (2020) 37:172–82. 10.14406/acu.2020.026

[15] Ministry of Health and Welfare Korea Health Industry Development Institute. Survey on the Use of Korean Medicine and Consumption of Herbal Medicine (2024). Available online at: https://www.mohw.go.kr/board.es?mid=a10411010100andbid=0019andact=viewandlist_no=1485229andtag=andnPage=1 (Accessed June 19, 2025).

[16] Song KJ. Strategy for revitalizing the Korean medical tourism industry: a focus on global case studies. Asia Pac J Converg Res Interchange. (2025) 11:585–97. 10.47116/apjcri.2025.01.44

[17] Labonté R Crooks VA Cerón Valdés A Runnels V Snyder J. Government roles in regulating medical tourism: evidence from Guatemala. Int J Equity Health. (2018) 17:150. 10.1186/s12939-018-0866-1 30236120 PMC6148768

[18] Almeman A. The digital transformation in pharmacy: embracing online platforms and the cosmeceutical paradigm shift. J Health Popul Nutr. (2024) 43:60. 10.1186/s41043-024-00550-2 38720390 PMC11080122

[19] Yu T Choi Y. Telemedicine centers as a venue for attracting international medical tourists. Korean Public Adm Rev. (2014) 28:133–67. 10.24210/kapm.2014.28.3.006

[20] Health Insurance Review and Assessment Service. Healthcare resource statistics: Korean medicine institution status (n.d.). Available online at: https://opendata.hira.or.kr (Accessed April 15, 2025).

[21] Lee J Ghang H. Healthcare utilization among foreign beneficiaries of the National Health Insurance program in Korea. BMC Health Serv Res. (2024) 24:1312. 10.1186/s12913-024-11698-2 39478541 PMC11526533

[22] Medical Korea. Search for foreign patient-attracting medical institutions (Traditional medicine clinics, Korean medicine hospitals) (n.d.). Available online at: https://www.medicalkorea.or.kr/korp/main.do (Accessed April 15, 2025).

